# Parkinson’s Disease: Potential Actions of Lithium by Targeting the WNT/β-Catenin Pathway, Oxidative Stress, Inflammation and Glutamatergic Pathway

**DOI:** 10.3390/cells10020230

**Published:** 2021-01-25

**Authors:** Alexandre Vallée, Jean-Noël Vallée, Yves Lecarpentier

**Affiliations:** 1 Department of Clinical Research and Innovation (DRCI), Hôpital Foch, 92150 Suresnes, France; 2 Centre Hospitalier Universitaire (CHU) Amiens Picardie, Université Picardie Jules Verne (UPJV), 80054 Amiens, France; valleejn@gmail.com; 3 Laboratoire de Mathématiques et Applications (LMA), UMR CNRS 7348, Université de Poitiers, 86021 Poitiers, France; 4 Centre de Recherche Clinique, Grand Hôpital de l’Est Francilien (GHEF), 6-8 rue Saint-Fiacre, 77100 Meaux, France; yves.c.lecarpentier@gmail.com

**Keywords:** WNT/beta-catenin pathway, lithium, Parkinson, inflammation, oxidative stress, glutamatergic pathway

## Abstract

Parkinson’s disease (PD) is one of the major neurodegenerative diseases (ND) which presents a progressive neurodegeneration characterized by loss of dopamine in the substantia nigra pars compacta. It is well known that oxidative stress, inflammation and glutamatergic pathway play key roles in the development of PD. However, therapies remain uncertain and research for new treatment is mandatory. This review focuses on the potential effects of lithium, as a potential therapeutic strategy, on PD and some of the presumed mechanisms by which lithium provides its benefit properties. Lithium medication downregulates GSK-3beta, the main inhibitor of the WNT/β-catenin pathway. The stimulation of the WNT/β-catenin could be associated with the control of oxidative stress, inflammation, and glutamatergic pathway. Future prospective clinical trials could focus on lithium and its different and multiple interactions in PD.

## 1. Introduction

Parkinson’s disease (PD) is one of the major neurodegenerative diseases (ND) with progressive neurodegeneration characterized by loss of dopamine in the substantia nigra pars compacta. PD originates in the brainstem or spinal cord of patients and PD remains asymptomatic for a very long time [1,2]. The aetiologies of PD are still unknown, but the presence of Lewy bodies (clusters of α-synuclein and ubiquitin proteins in neurons) has been observed from the early stages of the disease. PD presents symptoms of tremor, stiffness, bradykinesia, and postural instability. These symptoms only appear when most of the dopaminergic (DAergic) cells are lost in the substantia nigra pars compacta, expressing smooth and coordinated regulation of striatal motor circuits also lost [3]. Depression or Sleep behavior disorders with rapid eye movements (REM) are non-motor symptoms that can precede the onset of the disease. Aging is one of the main risk factors for neurodegeneration. Aging can deregulate the various pathways that control cellular homeostatic phenomena. Altered cells are the sites of many molecular abnormalities [4]. Several metabolic mechanisms, such as inflammation and oxidative stress, can lead to a neurodegenerative process. PD shows metabolic remodeling resulting in concomitant increase in oxidative stress and neuroinflammation [5,6]. In recent years, the WNT/β-catenin pathway has been considered one of the main pathway involved in PD [7,8]. The deregulation of the WNT pathway is considered as an initiating event of the PD [9].

Lithium, which was introduced in 1949, is the primary drug commonly used for the treatment of chronic mental illnesses, such as bipolar disorder, characterized by depressive and manic cycles. Several studies have shown that prophylactic lithium can reduce manic relapses, although its effectiveness was significantly lower in reducing depressive relapses [10]. Moreover, other studies have shown that lithium therapy may reduce suicides and suicide attempts in patients with mood disorders [11]. Recent advances seem to show that the benefits of lithium go beyond just treating mood. Neuroprotection against excitotoxicity or brain damage is another role of lithium [12]. However, on the other hand, several reports have shown that a high dose of lithium can induce irreversible neurotoxicity effects [13]. Clinical manifestations of lithium toxicity include renal dysfunction, neurologic dysfunction, gastrointestinal upset, cardiac manifestations and endocrine abnormalities [14]. Nevertheless, the ability of lithium to cause chronic kidney disease appears to be very low [15]. Lithium poisoning has a low mortality rate and persistent cerebellar neurological deficits are uncommon in uncomplicated acute poisoning [16]. Same observations were reported for appropriate lithium doses with rare cardiac manifestations [17], gastro-intestinal upset [18] and endocrine manifestations [19]. Moreover, low doses of lithium are correlated with lower side-effects [20] and two reviews advocate the use of lithium therapy despite its potential side effects [21,22]. This review focuses on the potential effects of lithium, as a potential therapeutic strategy, on PD and some of the metabolic mechanisms, oxidative stress, and inflammation, by which lithium provides its beneficial properties.

## 2. Parkinson’s Disease and Oxidative Stress

Numerous studies have shown an increase in oxidative stress (OS) in the emergence of PD [23,24,25,26,27,28,29]. PD is characterized by mitochondrial dysregulation as evidenced by increased production and subsequent release of reactive oxygen species (ROS) [30]. Decreased mitochondrial activity leads to cell damage and death by decreased energy production due to improved OS [31]. OS and mitochondrial deregulation are associated with dementia and cell death [32,33,34]. The development of PD is characterized by an improvement of these operating systems [5]. Decreased respiratory chain activity in PD substance nigra pars compacta is associated with increased ROS production and apoptosis [30,35,36].

As part of oxidative metabolism, oxygen free radicals can be physiologically produced by the human body. Within each mitochondria, during aerobic respiration, molecular oxygen (O_2_) is reduced to water molecules. By this phenomenon, O_2_, H_2_O_2_ and OH are produced by an oxygen leakage [6]. During infections, phagocytic cells are caused to generate high levels of NO, O_2_ and H_2_O_2_ to defend the organism to reduce infection. However, the free radicals produced can also destroy healthy cells in the body and can have a detrimental effect [37].

Many enzymes, including tyrosine hydroxylase, L-amino acid oxidase, and monoamine oxidase (MAO) are involved in dopamine metabolism and in the production of ROS [38]. The production of ROS is found to be exacerbated during inflammation. However, many signals can be confused with ROS. In microglia, the aggregation and accumulation of ROS-induced proteins may be the cause of the inflammation observed [39]. OS and inflammation are associated with four improved mechanisms in PD: reduction in 26S proteasomal activity, increase in iron levels, decrease in glutathione (GSH) levels, and impaired regulation of the mitochondrial complex I [40,41]. In the physiological stage, MAO produces H_2_O_2_. Conversely, during PD, H_2_O_2_ is transformed into hydroxyl radicals (OH) by iron via Fenton reactions. Thus, H_2_O_2_ and OH improve the operating system [42]. H_2_O_2_ and OH oxidize GSH in the cytosol [43] causing GSH leakage into PD. The release of GSH molecules leads to the conversion of glutamate and cysteine into glutamyl peptides and cysteinyl peptides. These produced peptides appear to be toxic to dopaminergic cells by increasing ROS production and binding to the cell membrane in dopaminergic neurons. These peptides decrease the activity of complex I of the mitochondria leading to the production of ROS and to OS [44]. Dopaminergic (DAergic) cells are unable to repair misfiled proteins in PD due to damaged seen in proteasomal systems [45]. OS stimulates the carbonylation of proteins, an irreversible and irreparable process. Carbonylation is a phenotype of cellular senescence leading to the aggregation of proteins. In PD, the aggregation of these proteins is one of the main pathological phenomena of nigrostriatal DAergic neurons. Thus, these aggregated proteins are at the origin of neuroinflammation and OS [46].

## 3. Parkinson’s Disease and Inflammation

Some evidence has shown that inflammation plays a major role in PD [47]. Inflammation can activate apoptosis pathways in dopamine cells in PD [48,49]. The interaction between PD and inflammation is mutual. Inflammation leads to the death of dopaminergic cells but on the other hand, the death of DAergic cells leads, in a vicious feedback, to inflammation [50]. Additionally, inflammation causes OS, forcing DAergic cells to activate their death signals [51]. Multiple inflammatory factors, including microglia, play a major role in the development of PD [52]. Activation of microglia stimulates their pro-inflammatory enzymes (such as inducible nitric oxide synthase and cyclooxygenase) and the release of pro-inflammatory cytokines (such as CXC motif chemokine 12 ligand (CXCL12), tumor necrosis α (TNF-α), interferon-γ (IFN-γ), interleukin (IL)-6 and IL-1β [53]. The NF-κB pathway has an important role in the secretion of these pro-inflammatory enzymes and cytokines within the microglia [54]. TNF-α activates the process of apoptosis by the death domain of the TNF-R1 receptor stimulating caspases 1 and 3 [55]. TNF-α causes a decrease in c-Rel-NF-κB. c-Rel-NF-κB possesses neuroprotective action by inhibiting apoptosis through B-cell lymphoma-extra-large pathway within dopaminergic neurons [54]. High levels of expression of CXCR4 (referred to fusin) and its CXCL12 ligand have been shown in PD. The complex formed by CXCR4-CXCL12 activates caspase 3, which is the cause of apoptosis leading to the death of neural cells [56,57]. The IFN-γ-IFNGR signaling complex phosphorylates the leucine-rich repeat protein kinase 2 (LRRK2) [58]. In microglia and dopaminergic neurons, LRRK2 binds with many cellular signaling. The activated LRRK2 protein inhibits the expression of c-Rel-NF-κB. Thus, inflammation is increased by insufficient c-Rel-NF-κB [59,60]. Stimulation of LRRK2 results in the formation of tau oligomers, which stimulate cell death signaling [61,62]. LRRK2 modulates the traffic of certain vesicles and its overexpression leads to the activation of inflammatory cytokines [63].

## 4. Parkinson’s Disease and Glutamatergic Pathway

Numerous studies have observed the association between glutamate-mediated excitotoxicity and PD [14,64]. PARK2 is an E3 ubiquitin ligase parkin-encoding gene and its mutation leads to PD. PARK2 mutations are associated with abnormal small parkin protein which is dysregulated and degraded. Parkin is involved in the stability of glutamatergic synapses. In PD, proliferation of glutamatergic synapses with excitotoxicity are involved by parkin mutations [65]. Glutamate excitotoxicity is correlated with the increase of Bax and p53 and the decrease of Bcl-2 [66]. The apoptosis attributed to glutamate is preceded by an upregulation in activator protein-1 (AP-1) due to the stimulation of c-Jun N-terminal kinase (JNK) and p38 mitogen-activated protein kinase (MAP kinase) and phosphorylation of c-Jun (Ser63) and p53 (Ser15) [67].

## 5. WNT/β-Catenin Pathway

The name WNT is derived from Wingless *Drosophila melanogaster* and its mouse homolog Int. WNT/β-catenin pathway is implicated in numerous signaling and regulating pathways, including embryogenesis, cell proliferation, migration and polarity, apoptosis, and organogenesis [68]. However, during numerous pathological states, the WNT/β-catenin pathway can be dysregulated, such as inflammatory, metabolic and neurological disorders, tissue fibrosis and cancers [69].

The WNT pathway is one of the member of the secreted lipid-modified glycoproteins family [70]. WNT ligands are produced by neurons and immune cells in the central nervous system [71]. Control of the WNT/β-catenin pathway implicates, embryonic development, cell fate, epithelial-mesenchymal transition (EMT), metabolism. WNT pathway dysregulation contributes to several neurodegenerative diseases including PD [6,72,73,74]. The WNT pathway has a main stage which is the β-catenin/T-cell factor/lymphoid enhancer factor (TCF/LEF). Accumulation of β-catenin in the cytoplasm is modulated by the destruction complex composed by AXIN, glycogen synthase kinase-3 (GSK-3β) and tumor suppressor adenomatous polyposis coli (APC). In absence of WNT ligands, this destruction complex leads to hyper-phosphorylation of the cytoplasmic β-catenin and involves its proteasomal degradation. In contrast, in their presence, the WNT ligands complex to Frizzled (FZL) and LDL receptor-related protein 5/6 (LRP 5/6) to stop the action of the destruction complex and to prevent the proteasomal β-catenin degradation. Β-catenin translocates to the nucleus to bind to TCF/LEF. This phenomenon stimulates the WNT target genes [75,76,77].

**Figure 1:**
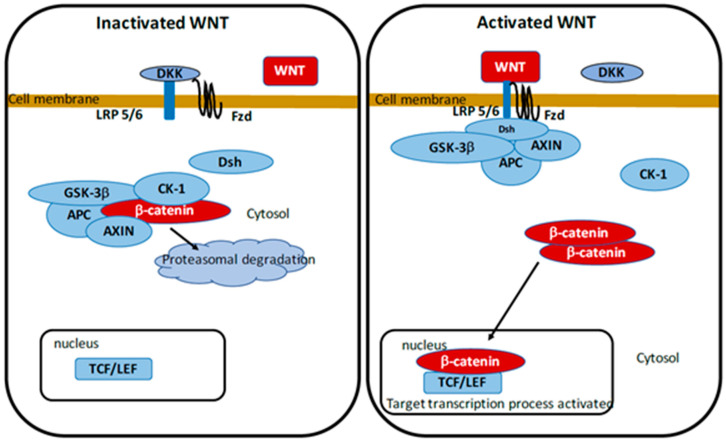
Activated and deactivated WNT pathway.

GSK-3β is one of the main inhibitors of the WNT/β-catenin pathway [78,79,80,81,82,83]. GSK-3β, an intracellular serine-threonine kinase, is a major controller and inhibitor of the WNT pathway [84]. It is implicated in the regulation of numerous pathophysiological pathways, including cell membrane signaling, cell polarity, and inflammation [85,86,87]. GSK-3β directly inhibits cytoplasmic β-catenin and stabilizes it leading to its nuclear migration. Inflammation is an age-related phenomenon associated with stimulation of GSK-3β activity and the diminution of the WNT/β-catenin signaling [88] (Figure 1).

### 5.1. Parkinson’s Disease and WNT/β-Catenin Pathway

Alteration of the WNT pathway is concomitant with the emergence of PD [7,8]. WNT signaling abnormalities are considered to be markers for the development of PD [9]. Multiple altered biological phenomena in PD are under the control of the WNT pathway, including microtubule stability, axonal function, and membrane trafficking [89,90]. The Frizzled-1/β-catenin pathway, directly controlled by WNT1, is responsible for the modulation of dopaminergic neuron-astrocyte crosstalk in the midbrain [91]. Under physiological conditions, LRRK2 binds to the WNT family and disheveled proteins (DSH) to inhibit the β-catenin destruction complex and to stimulate the WNT pathway [9]. In most cases, PD is an idiopathic form. However, familial PDs are often referred to PARK genes. Mutations in PARKs, encoding the leucine-rich repeat kinase 2 (LRRK2), have been shown to be a cause of familial forms of PD [92]. Decreased activity of the WNT pathway associated with decreased LRRK2-LRP5/6 binding affinity are caused by LRRK2 mutations [93]. Parkin is an E3 ubiquitin ligase encoded by the PARK2 gene. The genetic damage of parkin is responsible for the development of PD and works as repressors of β-catenin promoting ubiquitination and degradation of the latter [8]. DKK1 and GSK-3β are stimulated in PD [94]. PD mouse models exhibit crosstalk between inflammation, OS and WNT/β-catenin payhway [95].

### 5.2. WNT/β-Catenin Pathway and Oxidative Stress

Forkhead box class O (FoxO) transcription factors are main intracellular controllers of numerous metabolic signaling such as glucose production, and the cellular response to oxidative stress [96]. ROS is associated with the inhibition of the WNT pathway by diverting β-catenin from TCF/LEF to FoxO [97]. This leads to the accumulation and binding of β-catenin to FoxO as a cofactor, and in increasing FoxO transcriptional activity in the nucleus [98,99]. FoxO stimulates apoptotic genes [100,101,102]. FoxO3a stops the cell-cycle by stimulating of the production of the cyclin-dependent kinase inhibitor p27 kip1 and the inhibition of cyclin D1 expression [103,104]. The activation of FoxO leads to apoptosis induction [105]. However, the activation of the WNT pathway can downregulate FoxO3a in the cytosol to prevent the loss of mitochondrial membrane permeability, cytochrome c release, Bad phosphorylation, and activation of caspases which activates ROS production and oxidative stress [106].

### 5.3. WNT/β-Catenin Pathway and Inflammation

The stimulation of the WNT pathway cascade restrains inflammation and leads to the neuroprotection via interactions between microglia/macrophages and astrocytes [91,107]. Several studies have shown a negative crosstalk between WNT/β-catenin pathway and NF-κB signaling pathway, one of the main marker of inflammation [108]. The NF-κB transcription factor family belongs of five members in the cytosol under non-activated conditions: NF-κB 1 (p50/p105), NF-κB 2 (p52/p100), RelA (p65), RelB and c-Rel [109]. Β-catenin can complex with RelA and p50 to diminish the activity of the NF-κB signaling [110]. Moreover, by interacting with the PI3K, β-catenin inhibits the functional activity of NF-κB [111]. This inhibitory function of β-catenin on NF-κB activity has been observed in numerous cell types, such as fibroblasts, epithelial cells, hepatocytes and osteoblasts [108]. In parallel, the overactivation of GSK-3β leads to an inhibition of the β-catenin and then an activation of the NF-κB pathway [112]. The potential protective action of β-catenin was due to the activation of PI3K/Akt pathway and thus the reduction of TLR4-driven inflammatory response in hepatocytes [113]. NF-κB activation leads to the diminution of the complex β-catenin/TCF/LEF by the upregulation of LZTS2 in cancer cells [114]. DKK, a WNT inhibitor, was a target gene of the NF-κB pathway leading to a negative feedback to diminish the β-catenin signaling [115]. Activated Β-catenin inhibits the NF-κB -mediated transcription of pro-inflammatory genes. This effect is controlled by the GSK-3β. GSK-3β is a direct inhibitor of the β-catenin levels and an activator of the NF-κB signaling [116,117].

### 5.4. WNT/β-Catenin Pathway and Glutamatergic Pathway

β-Catenin activates EAAT2 an GS at the transcriptional level in progenitor-derived astrocytes through the activation of TCF/LEF [118]. The knockdown of β-catenin leads to the diminution of EAAT2 and GS expression in prefrontal cortex [119]. In astrocytes, the inhibition of β-catenin is associated with diminution of both EAAT2 and GS expression [120]. The dysregulation of the WNT/β-catenin pathway induces a glutamate excitotoxicity resulting in the increase of both inflammation and exudative stress [120].

## 6. Parkinson’s Disease: Interactions between WNT/β-Catenin Pathway and Lithium

**Figure 2:**
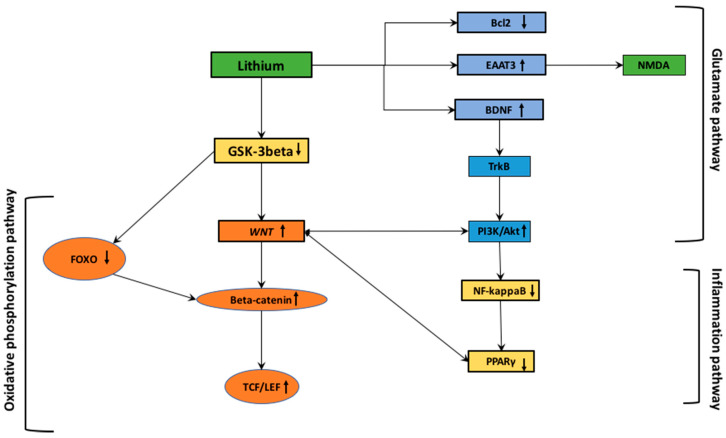
Lithium interactions with oxidative stress, inflammation and glutamatergic pathways.

A recent study has shown that mutant murine models of PD presented increased GSK-3β activity and thus its inhibition could be a treatment of perseverative behaviors (Figure 2).

Glycogen synthase kinase-3β (GSK-3β) is a serine/threonine kinase which is involved in numerous intracellular signaling pathways. Dysfunction of GSK-3β is involved in the pathogenesis of several diseases, including neuropsychiatric disorders [121]. GSK-3β is a regulator of several pathways such as inflammation, neuronal polarity or either cell membrane signaling [86]. GSK-3β downregulates the canonical WNT/β-catenin pathway by inhibiting β-catenin cytosolic stabilization and its translocation in the nucleus [93]. Moreover, numerous studies have observed a link between neuro-inflammation and the augmentation of the GSK-3β activity and in parallel the decrease of the WNT/β-catenin pathway and the protein kinase B (Akt) pathway [78].

Lithium at concentrations of 1 to 2 mM can inhibit GSK-3β activity [122,123,124]. Lithium reduces GSK-3β activity by increasing the inhibitory phosphorylation of GSK3β and through a direct activation of the Akt pathway. The activation of Akt modulates forkhead bow class O (FOXO), Bcl-2 associated death protein (Bad) (a pro-apoptotic protein of the Bcl-2 family) [125,126]. Therapeutic concentrations of the GSK-3β inhibitor lithium leads to the increase in β-catenin levels [127,128] and then promotes β-catenin transcriptional activity [129,130]. In brain of mouse, the over-expression of β-catenin levels mimic anti-depressant-like effects of lithium [131] while the knockout of β-catenin leads to a depression-like phenotype [132,133].

Several studies have shown the neuroprotective actions of lithium in models of PD [14,64,134,135,136,137,138,139]. Moreover, lithium inhibits GSK-3β thought its phosphorylation at Ser9 resulting in reduction of p-tau formation [140,141]. The inhibition of GSK-3β by lithium also result in the increase of β-catenin levels to enhance dopaminergic cell viability and then the reduction of α-synuclein [142,143]. PD patients show low CSF Aβ levels reflecting high brain Aβ deposition [144]. Aβ inhibits Akt and stimulates c-Abelson kinase (c-Abl) which increases p-tau formation through the activation of GSK-3β [6]. Lithium inhibits Aβ’s effects by inhibiting GSK-3β and cdk5 [145]. Degradation of intracellular proteins, including α-synuclein and p-tau, was influenced by autophagy-lysosomal pathway [146]. Akt/mTOR pathway can regulate authophagy [147].

The accumulation of α-synuclein leads to impaired autophagy and lysosomal functions [148]. Lithium can reduce α-synuclein aggregation by targeting autophagy through the Akt/mTOR pathway [149]. Enzymes have been proposed as potential targets of lithium action, such as inositol monophosphatase (IMPase), a family of structurally related phosphomonoesterases and the GSK-3β [149]. Lithium inhibits GSK-3β to reduce autophagy by activating the PI3K/Akt/mTOR pathway [150]. Lithium is a direct inhibitor of GSK-3β, which has a main role in oxidative stress through its inhibitory action on the WNT pathway. GSK-3β activity is regulated by site-specific phosphorylation. The activity of GSK3-β is upregulated by phosphorylation on the Tyr (216) residue, and conversely, phosphorylation on Ser(9) inhibits GSK-3β activity. In epidermal cells, ultraviolet B activated autophagy as a protective response and inhibited GSK3-β activation by simultaneously enhancing phosphorylation at Ser [151]. IMPase catalyzes the hydrolysis of inositol monophosphate (IP1) into free inositol required for the phosphoinositol signaling pathway [152]. Lithium affects this pathway by inhibiting IMPase, leading to free inositol depletion, which in turn decreases myo-inositol-1,4,5-trisphosphate (IP3) levels. Increased inositol or IP3 levels inhibit autophagy, which reverse lithium’s effect [153]. IP3 and the stimulation of its receptor have been seen to suppress autophagy [154]. The mitochondrial complex I inhibitors, 1-methyl-4-phenyl-1,2,3,6-tetrahydropyridine (MPTP), and rotenone were extensively used as neurotoxins to induce parkinsonian symptoms [155]. MPTP, mediated by GSK3-β, have been reported to enhance conversion of soluble α-synuclein to insoluble α-synuclein aggregates. Lithium administration can suppress MPTP activity and expression [156]. Lithium treatment ameliorates rotenone-induced toxicity in human neuroblastoma SH-SY5Y cells, which shows nuclear fragmentation and apoptosis [157]. A decrease in mitochondrial membrane potential, reduced reactive oxygen species generation and an increased number of lysosomes and autophagic vacuolar organelles was observed with 0.2 μm to 10 mM lithium administration [158]. Lithium administration protects against oxidative stress in the presence of α-synuclein A53T expression [134]. Moreover, lithium increases proteasomal activity which could explain the lithium-mediated reductions in α-synuclein and nitration/oxidization levels [159].

### 6.1. Lithium and Oxidative Stress

Energy and glucose metabolisms involved during oxidative stress are mainly controlled by the intracellular FOXO transcription factors (FOXO1, 3a, 4) [96]. The interaction between β-catenin and FOXO transcription factors promotes cell quiescence and cell cycle arrest. Β-catenin blocks its transcriptional complex with TCF/LEF through the interaction with FOXO-induced ROS [97]. Β-catenin does not translocate to the nucleus and thus accumulates in the cytosol leading to the inactivation of the WNT/β-catenin pathway [98,99]. Previous study has found that lithium can reduce FOXO3a transcriptional activity and can decrease the active FOXO3a level [160]. Thus, by inactivating GSK3-β, activating the WNT/β-catenin pathway and reducing the FOXO, lithium could participate to the reduction of oxidative stress.

Furthermore, numerous in vitro studies have shown that lithium administration could diminish hydrogen peroxide-induced cell death as well as obstruct lipid peroxidation and protein oxidation in cortical cells [134,161,162,163,164,165]. Moreover, lithium can act as an anti-oxidant by increasing CHS levels in neurons of rat dopaminergic N27 [134,162]. Moreover, the ability of lithium to act as an anti-oxidant was associate with the increase in GSH levels [134,162].

### 6.2. Lithium and Inflammation

Through the downregulation of GSK-3β activity and thus, the upregulation of the WNT/β-catenin pathway, the lithium administration could involve a diminution of the neuro-inflammation by acting on the NF-κB pathway (Figure 2). The stimulation of the WNT pathway cascade restrains inflammation and leads to the neuroprotection via interactions between microglia/macrophages and astrocytes [91,107].

Numerous studies have shown a negative crosstalk between WNT/β-catenin pathway and NF-κB signaling pathway [108]. The NF-κB transcription factor family belongs of five members in the cytosol under non-activated conditions: NF-κB 1 (p50/p105), NF-κB 2 (p52/p100), RelA (p65), RelB and c-Rel [109]. Β-catenin can form a complex with RelA and p50 to decrease the activity of the NF-κB signaling [110]. Moreover, by interacting with the PI3K, β-catenin inhibits the functional activity of NF-κB [111]. This inhibitory function of β-catenin on NF-κB activity has been observed in numerous cell types, such as fibroblasts, epithelial cells, hepatocytes and osteoblasts [108]. In parallel, the overactivation of GSK-3β leads to an inhibition of the β-catenin and then an activation of the NF-κB pathway [112]. The potential protective action of β-catenin was due to the activation of PI3K/Akt pathway and thus the reduction of TLR4-driven inflammatory response in hepatocytes [113]. NF-κB activation leads to the inhibition of the complex β-catenin/TCF/LEF by the upregulation of LZTS2 in cancer cells [114]. DKK, a WNT inhibitor, was a target gene of the NF-κB pathway leading to a negative feedback to diminish the β-catenin signaling [115].

A recent study has presented that the WNT pathway appeared to be one of the main mechanisms of action of lithium in adipose cells, and this interaction is done by the inhibition of PPARγ expression [166]. PPARs are ligand-activated transcription factors which bind PPRE (PPAR-response elements). PPARs are involved in numerous pathophysiological processes, such as cell differentiation, proteins metabolism, lipids metabolism, carcinogenesis [167,168], adipocyte differentiation, insulin sensitivity and inflammation [169,170]. PPARγ ligands, such as thiazolidinediones (TZDs), are able to decrease the inflammatory activity [171].

A negative crosstalk has been well described between PPARγ and the WNT pathway [172,173,174,175]. The PI3K/Akt pathway, which is positively induced by β-catenin [176,177], acts by phosphorylating GSK-3β to negatively regulate PPARγ expression [178]. PPARγ agonists decrease β-catenin expression by overactivating GSK-3β [179]. Moreover, PPARγ agonists stimulate Dickkopf-1 (DKK1) activity to diminish the canonical WNT/β-catenin pathway and then to decrease fibroblasts differentiation [180]. Moreover, PPARγ agonists stimulate GSK-3β to inhibit β-catenin expression [179].

### 6.3. Lithium and Glutamatergic Pathway

The administration of lithium was also associated with an influence on the levels of pro-apoptotic proteins (Figure 2). Bax, referred to as Bcl-2 associated C protein, is a key modulator promoting apoptosis by binding to and antagonizing Bcl-2 protein. The tumor suppressor protein, p53, targets Bcl-2 and Bax and then promotes growth arrest and cell death in response to cell damage [181].

Numerous studies have shown that the neuroprotective actions of lithium may be associated with increased levels of Bcl-2. Lithium therapy of cultured cerebellar granule cells stimulated the levels of mRNA and Bcl-2 protein, the level of Bcl-2/Bax protein level was increased by 5-fold after treatment during [66]. Stimulation of Bcl-2 expression induces neurogenesis within the hippocampus and entorhinal cortex in mice by increasing axon diameters and neurite growth on the CA3 area of the hippocampus and stimulates the myelination in the entorhinal cortex [182]. Lithium works by activating anti-apoptotic Bcl-2 levels and decreasing Bax expression [183]. The phosphorylation of Bcl-2 with serine 70 is essential for a complete anti-apoptotic action [184]. Several studies have shown that lithium possesses this ability [185]. Lithium decreases Bcl-2 dephosphorylation and caspase-2 stimulation by reducing expression of protein phosphatase-2A [185].

The excitotoxicity of glutamate has been strongly linked to the increase in the expression of Bax and p53 but also to the decrease in the expression of Bcl-2 [66]. The apoptotic process attributed to glutamate is preceded by the increased expression of activator-1 (AP-1) involved by stimulation of c-Jun N-terminal kinase (JNK) and mitogen-activated protein kinase p38 (MAP kinase) and phosphorylation of c -Jun and p53 [67].

By inhibiting GSK-3β activity, lithium acts as a powerful regulator of EAAT3 and therefore on the regulation of NMDA receptors [186]. In addition, a potential pathway could be the inhibition of presynaptic NMDA receptors and therefore the activation of postsynaptic AMPA receptors by the release of glutamate. This mechanism is followed by the activation of calcium influx and the secretion of brain-derived neurotrophic factor (BDNF). Thus, BDNF stimulates receptor tyrosine kinase B (TrkB) leading to neuronal survival and differentiation [187].

Activated BDNF-TrkB signaling leads to stimulate the Akt/mTOR pathway causing activation of the WNT/β-catenin pathway and improvement of synaptic proteins [188]. Low therapeutic level of lithium activates BDNF-TrkB signaling and then Akt/mTOR signaling to protect neurons from glutamate excitotoxicity [189]. Lithium inhibits excessive glutamate, NMDA receptor-mediated calcium influx into neurons, and reduces phosphorylation of the NR2B tyrosine subunit by Src/Fyn kinase [190].

## 7. Conclusions

Currently, few studies have investigated lithium as possible alternative therapeutic way to treat PD patients. Nevertheless, lithium could appear to be interesting against PD because of its potential inhibitory effect on oxidative stress, inflammation and glutamatergic pathway and this with few adverse effects at low doses. WNT/β-catenin pathway is decreased PD. Through, the stimulation of the WNT/β-catenin pathway, by the inhibition of GSK-3β, lithium, could be an innovative therapeutic way in PD. Future prospective studies could focus on lithium and its different and multiple interactions in PD. Autophagy is a “self-eating” mechanism which is induced to catabolize cellular substrates to generate energy. This process is responsible for the quality control of essential cellular components by purging cell of damaged organelles, such as peroxisomes and mitochondria, and by degrading aggregate-prone proteins [191]. The dysfunction of autophagy was observed in several neurodegenerative diseases, including Parkinson’s disease, Alzheimer’s disease, amyotrophic lateral sclerosis, Huntington’s disease [192]. These neurodegenerative disease also present a dysregulation of the WNT/β-catenin pathway [6]. Autophagy and WNT/β-catenin pathway are mainly associated [193] Thus, even if this article presents the interest of lithium in PD, lithium could be also of interest of other neurodegenerative disease [139].

## Data Availability

Not applicable.

## Abbreviations

GSK-3β: Glycogen synthase kinase-3β; LRP 5/6: Low-density lipoprotein receptor-related protein 5/6; NF-κB: nuclear factor kappaB; PPARγ: Peroxisome proliferator-activated receptor gamma; PI3K-Akt: Phosphatidylinositol 3-kinase-protein kinase B; TCF/LEF: T-cell factor/lymphoid enhancer factor; TNF-α: tumor necrosis factor alpha; PD: parkinson’s disease.
